# Gut Microbiome and Metabolites in Systemic Lupus Erythematosus: Link, Mechanisms and Intervention

**DOI:** 10.3389/fimmu.2021.686501

**Published:** 2021-07-15

**Authors:** Lingshu Zhang, Pingying Qing, Hang Yang, Yongkang Wu, Yi Liu, Yubin Luo

**Affiliations:** ^1^ Department of Rheumatology and Immunology, Rare Diseases Center, Institute of Immunology and Inflammation, Frontiers Science Center for Disease-related Molecular Network, West China Hospital, Sichuan University, Chengdu, China; ^2^ Department of Laboratory Medicine and Outpatient, West China Hospital, Sichuan University, Chengdu, China

**Keywords:** Systemic lupus erythematosus, gut microbiota dysbiosis, metabolites, SLE pathogenesis, SLE therapy

## Abstract

Systemic lupus erythematosus (SLE), often considered the prototype of autoimmune diseases, is characterized by over-activation of the autoimmune system with abnormal functions of innate and adaptive immune cells and the production of a large number of autoantibodies against nuclear components. Given the highly complex and heterogeneous nature of SLE, the pathogenesis of this disease remains incompletely understood and is presumed to involve both genetic and environmental factors. Currently, disturbance of the gut microbiota has emerged as a novel player involved in the pathogenesis of SLE. With in-depth research, the understanding of the intestinal bacteria-host interaction in SLE is much more comprehensive. Recent years have also seen an increase in metabolomics studies in SLE with the attempt to identify potential biomarkers for diagnosis or disease activity monitoring. An intricate relationship between gut microbiome changes and metabolic alterations could help explain the mechanisms by which gut bacteria play roles in the pathogenesis of SLE. Here, we review the role of microbiota dysbiosis in the aetiology of SLE and how intestinal microbiota interact with the host metabolism axis. A proposed treatment strategy for SLE based on gut microbiome (GM) regulation is also discussed in this review. Increasing our understanding of gut microbiota and their function in lupus will provide us with novel opportunities to develop effective and precise diagnostic strategies and to explore potential microbiota-based treatments for patients with lupus.

## Introduction

Systemic lupus erythematosus (SLE) is one of the most common systematic autoimmune diseases. It predominantly affects women of childbearing age and targets a diverse array of organs, including the skin, joints, kidney, lung, heart, and gastrointestinal tract, among others. Almost all SLE patients present with a large number of detectable autoantibodies against nucleic acid-(DNA or RNA)-associated proteins, with a particular emphasis on antinuclear antibodies (ANAs), anti-double stranded DNA (anti-dsDNA) antibodies, anti-Smith (Sm) antibodies, and anti-Sjögren’s syndrome-related antigens A and B (SSA/Ro and SSB/La, respectively) (1, 2). The heterogeneous clinical presentation of SLE indicates its complex aetiology. Although dozens of associated genomic variations have been identified by genomic analyses, the identified loci account for only 28% or less of disease heritability (3, 4). It is still puzzling how each variant functions in the pathogenesis of SLE. It is known that adaptive immune network dysregulation plays a significant role in the pathogenesis of SLE, in which abnormal T and B cell activation, self-antigen exposure and impaired apoptotic pathways contribute to a high frequency of autoantibody production in lupus (5, 6). Additionally, signal transducer and activator of transcription 4 (STAT4), interferon regulatory factor 5 (IRF5), IRF7, Toll-like receptor 7 (TLR7) and TLR9 are involved in pro-inflammatory cytokine production in monocytes/macrophages and type I interferon (IFN) production by dendritic cells (DCs), which induce and aggravate local and systemic inflammation in SLE (7, 8). However, the underlying molecular basis for the multifaceted manifestations of SLE is still indeterminate.

Environmental factors have also been postulated to be important triggers for initiating and promoting autoimmune reactions associated with SLE (9, 10). The microorganisms within our environment mainly include bacteria, protozoa, fungi, rickettsiae, viruses and helminths. The original research focusing on human-associated microbiota dates back to the 17th century (11), and the advent of high-throughput sequencing techniques represents a powerful tool to enhance our understanding of the biological activity of gut microbes (12–15). Within microbiome communities, *Bacteroidetes, Firmicutes, Actinobacteria* and *Proteobacteria* are four major phyla necessary to maintain a physiological gut bacterial ecosystem and homeostasis at distal sites. Gut microbes contribute to metabolic processes, the production of necessary secondary metabolites, and the shaping of host immunity (16). Within the previous two decades, expanding research has linked autoimmune diseases and intestinal bacterial composition changes (17–21). A fundamental role of the intestinal microbiome in the host is to help the mammalian intestinal immune system mature and protect the host from pathogen invasion by initiating pattern-recognition receptors (PRRs) in mucosal sites by expressing microorganism-associated molecular patterns (22). Changing the profile of metabolites is another important way in which gut bacteria affect host immune homeostasis. Regardless of whether the metabolites are solely derived from bacteria or drawn by co-metabolized bacteria and the host, their alteration commonly results in a local or systemic immune response change (23, 24). Elucidating the crosstalk between commensal microbiota, metabolites and the immune system may be crucial to understanding the immunopathology of SLE.

In this review, we aim to compile the available advances in understanding how gut microbiota functionally affect the development of various autoantibodies and how bacteria-derived metabolites contribute to inflammation and the interaction between intestinal microbiota and medication in the pathogenesis of SLE. Moreover, we also present evidence- or hypothesis-based perspectives for new therapeutic strategies for SLE.

## The Gut Microbiota in SLE

Mounting evidence suggests that a wide range of autoimmune conditions have characteristic patterns of gut microbiome dysbiosis, such as inflammatory bowel disease (IBD) (18), diabetes (19), multiple sclerosis (25) and rheumatoid arthritis (RA) (21). A link between gut microbiota populations and SLE has also been proposed recently.

### The Link Between GM Dysbiosis and SLE in Humans

Since gut symptoms are not specific and typical in SLE patients, the link between gut bacteria and SLE development was neglected by the rheumatologists or scientists before. This streak was broken by Hevia *et al.* in 2014, who reported that patients with lupus presented restricted gut microbiome diversity compared with 20 healthy counterparts (26). In addition, patients with SLE also have altered structure and composition of the gut microbiota, and the proportions of bacteria at multiple levels differ from healthy individuals, including the enrichment of *Proteobacteria, Bacteroidetes* and *Actinobacteria* and a lower proportion of *Firmicutes* compared with healthy controls (27). The *Firmicutes/Bacteroides (F/B)* ratio is contradictory in some studies, which is probably because of the differences in the enrolment criteria of patients, including race, sex ratio, active disease duration, and immune therapy methods (**Table 1**).

**Table 1:** Microbiota alternation in patients with SLE.

Human subjects (n)	Region	Bacteria in SLE	Other	Reference
SLE (20) vs HC (20)	Spain	**Phyla:** *Firmicutes/Bacteroidetes* ratio↓, *Bacteriodetes* ↑.		(26)
SLE (45) vs HC (48)	China	**Phyla:** Firmicutes/Bacteroidetes ratio**↓,** *Firmicutes* ↓;*Bacteriodetes* ↑; **Family:** *Rhodococcus, Eggerthella, Klebsiella, Prevotella, Eubacterium, Flavonifractor* and *Incertae sedis*↑; *Dialister, Pseudobutyrivibrio*↓.		(27)
SLE (61) vs HC (17)	USA	**Species:** *Ruminococcus gnavus*↑.	Anti-RG antibodies correlated with SLEDAI score and active nephritis.	(28)
SLE (92) vs HC (217)	China	**Phyla:** *Bacteroidetes, Proteobacteria*, and *Actinobacteria* ↑, *Firmicutes*↓; **Family :** *Bacteroidaceae,Streptococcaceae*↑; *Ruminococcaceae, Veillonellaceae, Lachnospiraceae*↓; **Genus:** *Ruminococcus*, Kl*ebsiella, Erysipelot*richaceae↑, *Faecalibacterium*↓.	*Ruminococcus* correlated with the absolute counts of Tregs in peripheral blood.	(29)
SLE (30) vs HC (965)	Netherlands	**Phyla:** *Firmicutes/Bacteroidetes* ratio↓, *Bacteroidetes, Proteobacteria*↑; **Genus:** Bacteroides, *Alistipes*↑; **Species:** *Bacteroides vulgatus, Bacteroides uniformis*, *Bacteroides ovatus* and *Bacteroides thetaiotaomicron↑.*		(30)
SLE (14) vs HC (17)	USA	**Phyla:** *Firmicutes/Bacteroidetes* ratio was not different, *Proteobacteria*↑; **Genus:** *Odoribacter* ↓, *Blautia* ↑.		(31)
SLE (12) vs HC (22)	USA	**Genus:** *Lactobacillus* spp.↑.		(32)
SLE (16) vs HC (11)	USA	**Phyla:** *Firmicutes/Bacteroidetes* ratio↓.	Human Ro60 autoantigen–specific CD4 memory T cell clones from lupus patients react to *Bacteroides thetaiotaomicron* containing Ro60 orthologs.	(33)
SLE (117) vs HC (115)	China	**Species series A** *: Clostridium species* ATCC BAA-442*, Atopobium rimae, Shuttleworthia satelles, Actinomyces massiliensis, Bacteroides fragilis, Clostridium leptum*↑; **Species series B** *: Lactobacillus salivarius, Ruminococcus gnavus, Clostridium nexile, Olsenella uli, Actinomyces johnsonii, Staphylococcus aureus, Enterococcus avium*↑.	All species in series A were reduced after treatment and most were more closely associated with oral cavity species in SLE vs healthy subjects. *Clostridium species* ATCC BAA-442 and *L.salivarius* abundances positively correlated with SLEDAI score.	(34)
SLE (21) vs HC (10)	China	**Phyla:** *Firmicutes/Bacteroidetes* ratio↑, *Bacteriodetes*↓.		(35)

HC, healthy controls; RG, Ruminococcus gnavus; SLEDAI, SLE disease activity index. The bold type indicates the classification level of the bacteria.

Interestingly, SLE-related gut microbiota share some similarities across geographical locations, as observed in a study examining SLE subjects from Spain and China. Both groups showed an enrichment of *Prevotellaceae* when compared to their healthy counterparts (27). However, a depletion of *Lachnospiraceae* and *Ruminococcaceae* species and a significant increase in *Bacteroidaceae* were detected in patients from Spain compared to those from China (26). Another microbial population of interest in autoimmune disorders is *Prevotella.* A flourishing dietary structure and genetic background may contribute to intestinal disturbances in different populations with autoimmune disorders. Interestingly, the genera *Rhodococcus, Eggerthella, Klebsiella, Prevotella, Eubacterium, Flavonifractor* and *Incertae sedis* are significantly prevalent in SLE patients, while the genera *Dialister* and *Pseudobutyrivibrio* are considerately reduced (**Table 1**) (27). At the species level, *Ruminococcus gnavus* (RG) of the *Firmicutes* phylum thrives in the gastrointestinal tract and promotes gut barrier impairment, which is especially prominent in SLE patients with renal involvement. Moreover, species richness diversity was directly paralleled with the SLE disease activity index (SLEDAI) (28). Furthermore, anti-RG antibodies are positively correlated with the SLEDAI score and anti-native DNA levels but negatively correlated with C3 and C4 levels (**Table 1**) (28).

### The Effect of GM Dysbiosis on Immune Cells in SLE

More recent detailed studies point to the effects of GM dysbiosis in SLE patients on immune cell phenotype switching and functions, which further proves their importance in immunological tolerance breaks in SLE. T cells, B cells and plasmacytoid dendritic cells (pDCs) are pivotal immune cells implicated in SLE pathogenesis (10). *Prevotella* cultures are strongly associated with augmented T helper type 17 (Th17)-mediated mucosal inflammation and the production of interleukin (IL)-8, IL-6 and CCL20 through the stimulation of epithelial cells, leading to local or systemic chronic inflammation (36). Some *Prevotella* strains play an important role in intestinal disturbance of RA (37, 38), metabolic syndromes (39, 40), and IBD (41). Another study found that patients with SLE had a significantly lower abundance of *Ruminococcaceae* at the family level but a higher percentage of *Ruminococcus* at the genus level. Notably, the proportion of *Ruminococcus* was positively correlated with the absolute count of Treg lymphocytes but not with the number of Th1, Th2 and Th17 cells, assuming that immune cells in circulation may respond to bacterial dynamics in the intestinal site (**Table 1**) (29). A report from Chen *et al.* pointed out that intestinal symbiont colonization not only enriched antibacterial specificities in the earliest B cell repertoires in the small intestine but also primed reactive IgA responses and enhanced systemic IgG responses to bacterial antigens (42). These findings present the possibility that gut microbiota dysbiosis in SLE might be responsible for breaking immunological tolerance to autoreactive T cell or B cell clones in the gut. In fact, an increasing number of studies point to the correlation of autoimmune antibodies with disturbed intestinal bacteria in SLE, which makes this hypothesis more credible.

### The Effects of GM Dysbiosis on Lupus-Related Antibody Production

The production of dozens of autoimmune antibodies is a major characteristic of systemic autoimmune diseases; however, the mechanisms of their overexpression remain unclear. ANAs are a hallmark feature of SLE, systemic sclerosis (SSc), and polymyositis, making them the most sensitive and effective biomarker for the diagnosis of a systemic autoimmune disease. Almost all lupus patients produce high titres of ANA for years prior to the appearance of clinical symptoms.

Anti-DNA antibodies were originally described in patients with bacterial infections in the late 1930s (43). Since 1957, anti-DNA antibodies have been discovered in lupus patients (44–46), and antibodies directed against dsDNA have been associated with SLE disease severity. Anti-dsDNA antibodies are specific autoantigens targeted in SLE with a high SLEDAI. The levels of anti-dsDNA antibodies are related to the severity of nephritis (47, 48) and sufficient for SLE classification in both the 2012 Systemic Lupus International Collaborating Clinics (SLICC) criteria (49) and the 2019 European League Against Rheumatism (EULAR)/ACR classification criteria (50, 51). Patients with lupus have intestinal dysbiosis accompanied by reduced diversity richness of microbiota compared to healthy controls. Some species like the relative abundance of *Bacteroides vulgatus*, *Bacteroides uniformis* and *Bacteroides ovatus* was significantly higher in SLE patients than in population controls (**Table 1**) (30). A recent study reported that lupus patients have impaired intestinal barrier integrity and possess a striking fivefold increase in the abundance of intestinal *Ruminococcus gnavus* (RG). Moreover, serum anti-RG strain-restricted antibodies correlated directly with the SLEDAI score and anti-native DNA levels but inversely with C3 and C4 in lupus patients (28). The greatest titre of serum anti-RG strain-restricted antibodies was detected in those with active lupus nephritis (including class III and IV) (**Table 1**) (28). The transplantation of faeces from SLE mice to GF recipients resulted in an enhanced intestinal immune response and upregulated expression of antibody titres against dsDNA in GF mice in contrast to those that received B6 faeces (52).

Antiphospholipid syndrome (APS) is an autoimmune thrombophilia characterized by the presence of vascular thrombosis or pregnancy complications and anti-phospholipid antibodies, including lupus anticoagulant (LA), anticardiolipin antibody (aCL) and anti-beta 2 glycoprotein 1 (anti-β2 GPI) (53). APS was first described in lupus patients (54), and approximately 40% of lupus patients possess these antibodies (55). The presence of higher concentrations of IgA anti-β2GPI antibodies is associated with disease prevalence or morbidities involving the gastrointestinal system, pulmonary system, or skin and a higher risk of thromboembolic events, especially among lupus patients (56). A recent finding reported that the human gut commensal *Roseburia intestinalis* (*R. int*), a widely prevalent bacterium in the host, contains amino acid sequences that are highly homologous to sequences found in B and T cell epitopes within β2GPI (57). Significantly higher expression of faecal calprotectin was also identified in APS patients, indicating that subclinical intestinal and peripheral inflammation facilitates mucosal autoimmune responses to commensal bacteria, leading to systemic spread through cross-reactivity. Moreover, patients with APS experience the greatest levels of antibodies binding to a mimotope in a bacterial DNA methyltransferase expressed by *R. int* (R. int DNMT), and in these individuals, the induction of anti-R. int DNMT IgG surpassed that observed in healthy controls. Mice immunized with *R. int* lysates triggered a substantial rise in anti-rhβ2GPI autoantibody titres compared to *B. theta*- or sham-immunized controls *in vivo*. Furthermore, transplantation of *R. int* into a mouse model of spontaneous APS (NZW x BXSB) F1 triggered the development of anti-human-β2GPI antibodies and thrombotic events (57).

Taken together, the above studies indicate a close association between commensal microbiota and lupus-related antibodies. However, the implications of some specific bacteria in terms of immunological tolerance to self-antigens were not investigated and could only be suspected.

## Gut Microbiota of Murine Lupus Models

### Gut Microbiome Dysbiosis and SLE Development—Evidence From Lupus Animal Models

Emerging investigations in rodent models have confirmed the role of gut microbiota in the development of SLE. In an earlier study, Maldonado *et al.* did not observe a clinical difference between germ-free (GF) MRL/lpr mice and conventional mice. This difference appeared only when they were fed an ultrafiltered antigen-free diet (58). However, Johnson *et al.* then reported a significant and dynamic change in the gut microbiota communities before and after the onset of disease in lupus-prone (SWR × NZB) F1 mice (**Table 2**) (59). Interestingly, these female mice displayed more severe clinical lupus symptoms, associated with a higher abundance of a group of *Lactobacilli* in the gut compared to the male mice. In line with this finding, Zhang *et al.* observed an overrepresentation of *Lachnospiraceae* in female mice and an earlier onset of and/or more severe lupus symptoms (**Table 2**) (60), suggesting that the disturbed intestinal flora contributes to lupus development in a sex-dependent manner in mice.

**Table 2:** Microbiota alternation in lupus-related mouse models.

Model	Bacteria in disease	Other	Reference
TC *vs.* C57BL/6 mice	**Class:** *Erysipelotrichia, unidentified Actinobacteria*↑; *Bacilli*↓; **Order:** *Erysipelotrichales*↑; *Lactobacillales*↓; **Family:** *Erysipelotrichaceae*↑; *Rikenellaceae, Lactobacillaceae*↓; **Genus:** *Turicibacter*↑; *Alistpes*, *Lactobacillus*↓.	Feces transplantation from TC mice to C57BL/6 germ-free mice induces anti-dsDNA antibodies and innate immune response.	(52)
(SWR × NZB)F1 mice AW *vs.* NW	**Phyla:** *Firmicutes/Bacteroidetes* ratio was not different; **Family:** *Rikenellaceae*↓; **Genus :** *Turicibacter* spp.↑; **Species:** *Lactobacillus reuteri*↑.	AW-fed mice have accelerated nephritis	(59)
MRL/lpr *vs.* MRL mice	**Family:** *Lachnospiraceae*, *Ruminococcaceae, Rikenellaceae*↑; *Lactobacillaceae*↓.	Lupus accelerated/more severe in females also associated with *Lachnospriraceae*↑.	(60)
NZB/W F1 mice	**Genus:** *Clostridium*, *Dehalobacterium*, *Lactobacillus*, *Oscillospira*, *Dorea, Bilophila, AF12* and an unnamed genus within the family *Ruminococcaceae↑*; **Species:** *Akkermansia muciniphila* and *a* species within the genus *Anaerostipes*↓.	Treatment with Dex decreases *Lactobacilli* which might be associated with progressing disease.	(31)
MRL/lpr *vs.* MRL mice	**Order:** *Lactobacillales*↓.	*Lactobacillus* can correct the leaky gut, improve renal function and increase the survival of female lpr mice.	(61)
TLR7.1 Tg *vs.* WT C57BL/6 mice	**Species:** *Lactobacillus reuteri↑.*	*Lactobacillus reuter* increased plasmacytoid dendritic cells and interferon signaling.	(32)
(NZW × BXSB)F1 *vs.*C57BL/6 mice	**Species:** *Enterococcus gallinarum*	*Enterococcus gallinarum* translocated from the intestine to the liver; vaccination with heat-killed *E. gallinarum* decreased serum autoantibodies and increased survival. *E. gallinarum* also found in liver biopsies of some SLE, but not control patients.	(62)
MRL/lpr *vs.* MRL/MpJ mice	**Genus :** *Blautia* **↑,** *Desulfovibrio*↓; **Species:** *Ruminococcus torques* ↑		(34)
TC *vs.* C57BL/6 mice	**Genus:** *Prevotellaceae, Paraprevotella* and *Lactobacillus*↑.	Tryptophan metabolism was altered in TC mice; *Prevotella* species is associated with tryptophan metabolism. High tryptophan diet exacerbated and low tryptophan diet mitigated disease in TC mice and both diets altered microbiota and its ability to transfer disease to C57BL/6 germ-free mice.	(63)
(SWR × NZB)F1 mice Female *vs.* Male	**Genus:** *Bacteroides*, *Parabacteroides*↑; *Dysgonomonas*↓.	Gender specific difference in gut microbiota appears at adult age.	(64)

AW, acidic pH water; NW, neutral pH water; Dex, dexamethasone; TLR7, Toll-like receptor 7; WT, wild-type; TC, B6.NZM-Sle1^NZM2410/Aeg-^ Sle2^NZM2410/Aeg^Sle3^NZM2410/Aeg^/LmoJ. The bold type indicates the classification level of the bacteria.

### Potential Mechanisms Linking Intestinal Bacteria to SLE

#### Specific Bacterial Populations and Pathways Linked to the Microbiota and SLE

The roles of *Lactobacillus* species in the pathogenesis of lupus-prone mice have been the most studied. *Lactobacillus* species are known for their capacity to harvest nutrients and generate energy but are commonly used as probiotic agents to regulate immune and anti-inflammatory responses (65). NZB/W F1 mice tend to have more diverse microbiota and enriched groups of *Lactobacilli* after dexamethasone treatment (**Table 2**) (31). The probiotics *Lactobacillus rhamnosus* and *Lactobacillus delbrueckii* were shown to downregulate the expression of miR-155 and miR-181a, which is positively correlated with the SLEDAI and kidney involvement in the PBMCs of SLE patients (66, 67). *Lactobacillales* has been reported to be markedly depleted in MRL/lpr mice, and increasing *Lactobacillales* can correct the leaky gut, promote IL-10, improve renal function, and prolong mouse survival (**Table 2**) (61). However, in 2019, Zegarra-Ruiz *et al.* identified an enrichment of another *Lactobacillus* species, *Lactobacillus reuteri (L. reuteri)*, in lupus models. Unlike the other *Lactobacillus* species mentioned above, *L. reuteri* can worsen autoimmune manifestations by engaging in type I interferon pathways (**Table 2**) (32). Another interesting report is from Manfredo Vieira *et al.* Oral administration of antibiotics suppressed the growth of intestinal gram-positive bacterium *Enterococcus gallinarum* (*E. gallinarum*), leading to the intestinal epithelial tight junction decrease, the lupus-related autoantibodies reduction and the lifespan extension of lupus-prone mice. Vaccination with heat-killed *E. gallinarum* decreased serum autoantibodies and increased survival of the lupus-prone mice. *E. gallinarum* also found in liver biopsies of some SLE, but not control patients (**Table 2**). Moreover, the translocation of *E. gallinarum* from the intestine to the liver triggered innate autoimmunity by potently inducing IFN-α production by plasmacytoid dendritic cells and Th17 cell expansion through activation of the AhR pathway (62).

A mouse model of SLE, B6. NZM-Sle1^NZM2410/Aeg-^ Sle2^NZM2410/Aeg^Sle3^NZM2410/Aeg^/LmoJ mice, known as TC (SLE) mice, display anti-dsDNA antibodies in serum and immune cell disturbances (68). TC mice also exhibited restricted richness and diversity of microbiota compared with C57/B6 mice (B6 mice). *Erysipelotrichia, unidentified Actinobacteria, Erysipelotrichales, Erysipelotrichaceae, Turicibacter* were enriched in TC mice and *Bacilli, Lactobacillales, Rikenellaceae, Lactobacillaceae, Alistpes, Lactobacillus* were microbial makers in B6 mice. GF mice administered SLE faeces had similar microbiota to their donors and showed significantly increased serum anti-dsDNA antibodies three weeks after faecal microbiota transplantation (FMT). Moreover, these mice possessed significantly higher percentages of plasma blasts, plasma cells and CD4^+^RORγt^+^ T cells but presented with a decreased proportion of Treg cells in the spleen, indicating that gut microbiota from TC mice has pivotal functions in immune response regulation (**Table 2**) (52). As the type-I IFN signalling pathway is critical in the pathogenesis of SLE (69), gut microbiota-derived IFN transcription may further enhance disease development by initiating downstream signalling of IFN (52).

#### Molecular Mimicry

**Figure 1:**
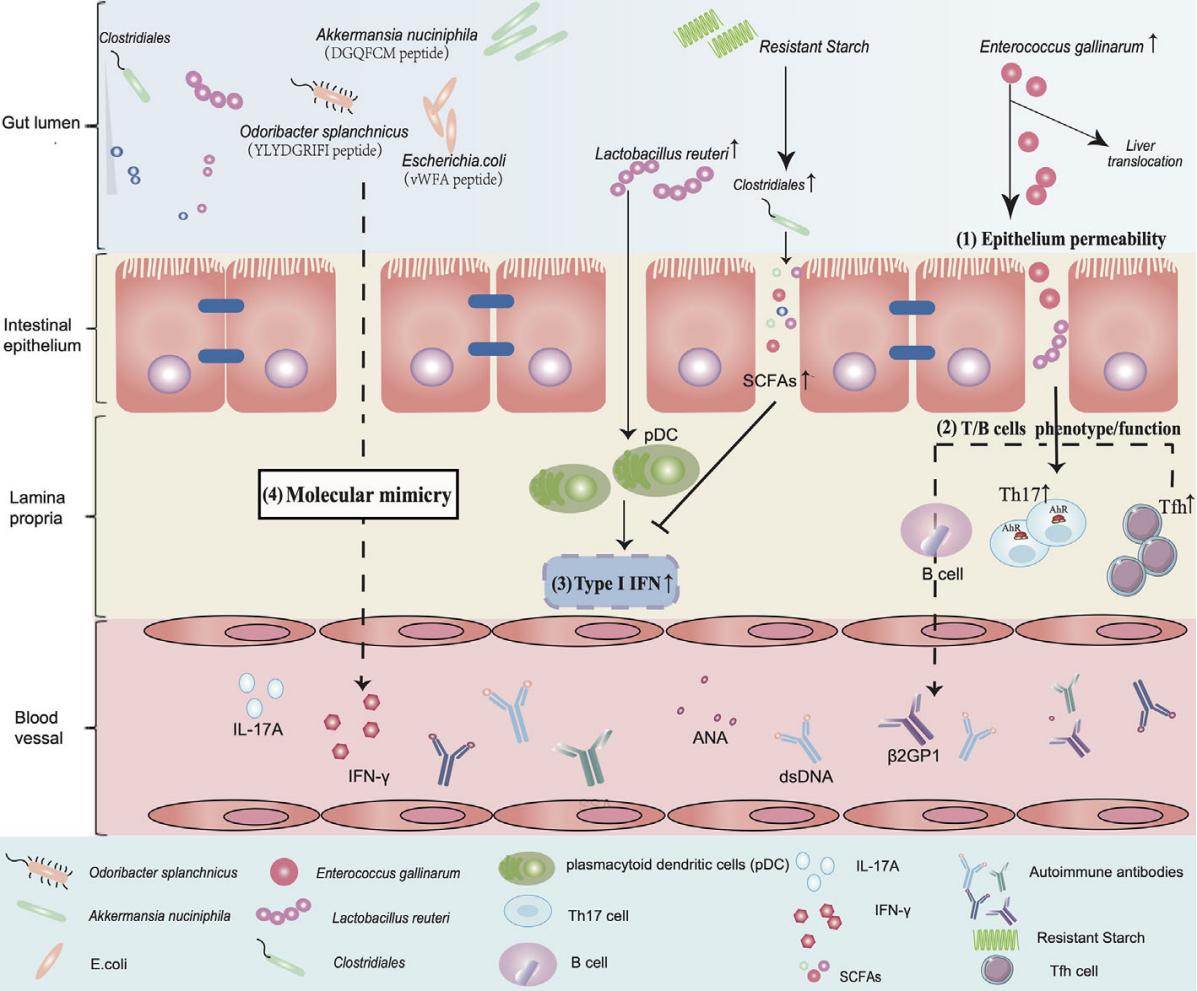
An overview of the implication of gut microbiota in the aetiology of SLE. (1) Epithelium permeability: *Enterococcus gallinarum* induces an increase in epithelial permeability and translocates from the gut to the liver. (2) T/B cell phenotype/function: This triggers autoimmunity by upregulating the function of pDCs and Th17 cells and promotes the production of autoimmune antibodies such as dsDNA and β2GP1 in systemic circulation. (3) Type I IFN: *Lactobacillus reuteri* enriched in a lupus model can worsen autoimmune manifestations by engaging in type I interferon pathways. Dietary resistant starch feeding of mice increases *Clostridiales* abundance and promotes SCFA production to suppress type I IFN production. (4) Molecular mimicry: The peptide “YLYDGRIFI” of *Odoribacter splanchnicus* significantly increases IFN-*γ* and IL-17A expression in PBMCs of a subgroup of anti-Sm-positive SLE patients. The peptide “DGQFCM” from *Akkermansia nuciniphila* is capable of mimicking the extracellular part “DGQFCH” of human Fas and binds to IgG produced by memory B cells from SLE patients. Bacterial vWFA proteins expressed by *E. coli* can activate Ro60 reactive T cells.

Another possible mechanism linking the intestinal microbiota to SLE pathogenesis is molecular mimicry (**Figure 1**). This hypothesis was addressed in two independent reports by Greiling *et al. *(33) and Chen *et al. *(34). Antibodies against Ro60/SSA/TROVE2, an evolutionarily conserved RNA-binding protein, are present in more than 50% of lupus patients (70). Ro60 is upregulated following ultraviolet (UV) irradiation (71, 72), which may trigger and flare the lupus disease process (73). Evidence from bacterial and animal cells supports a function for Ro60 in the mitigation of the environmental stress response (74). As a highly conserved RNA-binding protein, orthologues of Ro60 are found in bacteria that reside at a variety of anatomical locations, including the skin, oral tract, and gut (74, 75). Cross-reactivity between Ro60 and EBV nuclear antigen-1 (EBNA-1) protein produced after EBV infection has been proposed as a mechanism for the progressive development of anti-Ro60 antibody in lupus patients (76). It has been reported that bacterial vWFA proteins expressed by *E. coli* can activate Ro60 reactive T-cell hybridomas in HLA-DR3 transgenic mice, demonstrating that peptides derived from commensal microbiota might be implicated in the initiation of autoimmune responses to Ro60 (77). Greiling *et al.* further demonstrated functional cross-reactivity between human Ro60 autoantigen-specific memory CD4 T cell clones from lupus patients and Ro60 orthologues from mucosal Ro60-containing species, including *Bacteroides thetaiotaomicron*, *in vitro* (**Table 1**). Mono-colonization of GF wild-type C57Bl/6 mice with the common gut commensal *B. thetaiotaomicron* resulted in anti-Ro60 T and B cell responses *in vivo *(33). Another recent study also supports this molecular mimicry hypothesis in SLE. While certain species were found to be enriched in SLE patients, including *Clostridium species ATCC BAA-442, Atopobium rimae, Shuttleworthia satelles, Actinomyces massiliensis, Bacteroides fragilis*, and *Clostridium leptum* but reduced after treatment. *Lactobacillus salivarius, Ruminococcus gnavus, Clostridium nexile, Olsenella uli, Actinomyces johnsonii, Staphylococcus aureus, Enterococcus avium* were enriched in patients with lupus nephritis. Fecal *Clostridium species ATCC BAA-442* and *Lactobacillus salivarius* correlated positively with SLEDAI. Moreover, the enrichment of oral species including *Actinomyces massiliensis, Shuttleworthia satelles, Clostridium* sp. *ATCC BAA-442, Bacteroides fragilis*, and *Clostridium leptum* in SLE suggests transmission rate of salivary microbes to intestine in SLE condition (**Table 1**). Genera *Desulfovibrio* was decreased while *Blautia* and *Ruminococcus torques* were increased in the gut of MRL/lpr (**Table 2**). In addition to the differences in the oral and gut microbiomes between healthy individuals and SLE patients, researchers also identified two peptides, “YLYDGRIFI”, of the IS66 family transposase from *Odoribacter splanchnicus* and “DGQFCM”, from *Akkermansia muciniphila* (**Table 1**) (34). The peptide “YLYDGRIFI” was capable of upregulating IFN-*γ* and IL-17A production from the PBMCs of a subgroup of anti-Sm-positive SLE patients in an HLA-DR-dependent manner. The peptide “DGQFCM” can mimic the extracellular part “DGQFCH” of human Fas and bind to IgG produced by memory B cells from SLE patients but not those of healthy controls (34).

Collectively, these investigations indicate microbial dysbiosis in the gut of SLE patients that results in reduced bacterial diversity and decreased proportions of protective commensal species. The ‘leaky’ gut, Th17 cell expansion, IFN-*γ* pathway activation and molecular mimicry associated with gut microbiota dysbiosis play an inconvenient role in the pathogenesis of SLE. Despite these findings, it remains unclear whether translocation of microbial species or their products from the gut into other organs occurs and to what extent sex-specific differences occur in human SLE patients. Addressing these questions may provide new insights into the in-depth mechanisms that lead to the onset of SLE and the differences in the clinical presentation of SLE.

## Metabolic Perturbations Linked to SLE Development

The nature of the intestinal microbiota to coevolve with the host is essential for energy capture by digestion enzymes that do not exist in the human genome. Bacterial metabolites have significant effects on the metabolic pathways of dietary carbohydrates, fat, protein and vitamins (78). A mounting array of studies has revealed that patients with SLE have abnormal metabolism compared to the general population (79–81). These metabolite changes are indicated in amino acid metabolism, glycometabolism, lipid metabolism and carbohydrate metabolism. Here, we focus on current research evaluating the disturbance or roles of lipids, short-chain fatty acids and amino acids in the pathogenesis of lupus.

### Lipoprotein Metabolism

Lipoprotein particles are synthesized in the liver and intestine and are composed of lipids (such as phospholipids, cholesterol and triglycerides) and apolipoproteins (82). The gut microbiota has the ability to modulate dietary lipid composition, digestion, and absorption, potentially altering intestinal lipoprotein formation (83) (84). Available evidence suggests that patients suffering from SLE are more prone to develop lipid profile disorders in both serum and faeces (85) (35). Enriched lipids accounted for 65% of all changed metabolites in serum from SLE patients. In addition, some bile acids, including deoxycholic acid, GCA, isohyodeoxycholic acid and arachidonic acid, were significantly correlated with the SLEDAI score. Increased levels of primary bile acids, including cholic acid (CA), glycocolic acid (GCA), taurocholic acid (TCA) and glycochenodeoxycholic acid (GCDCA), were also observed in faeces from SLE patients, and they showed fine power to predict the SLEDAI score in a multiple linear regression model (35). It is worth noting that bile acids are important signalling molecules and exert functions through activation of farnesoid X receptor (FXR), GPCR (also known as TGR5) and vitamin D receptor (VDR) (86). FXR has been reported to be downregulated in SLE patients as well as MRL/lpr lupus models with liver dysfunction. Administration of the FXR agonist chenodeoxycholic acid (CDCA) suppresses inflammatory cytokines such as TNF-α, IFN-γ, and IL-6 in mice (87). A recent study also identified the modulatory effect of bile acids on gut immunity. Derivatives of lithocholic acid (LCA), 3-oxoLCA and isoalloLCA can inhibit the differentiation of Th17 cells by directly binding to the key transcription factor retinoid-related orphan receptor-γt (RORγt). Moreover, they enhanced FOXP3 expression by producing mitochondrial reactive oxygen species (mitoROS), leading to the expansion of Treg cells (88). Another study demonstrated that secondary bile acids can activate RORγ^+^ regulatory T cells *via* VDR in the colonic lamina propria of mice (89). In a separate study, researchers identified another type of bile acid, secondary BA 3β-hydroxydeoxycholic acid (isoDCA), which can induce peripheral Treg (pTreg) generation in the large intestine. The microbial fermentation of isoDCA can confer upon an anti‐inflammatory profile in dendritic cells to maintain intestinal homeostasis (90). To date, there is still a lack of systemic and mechanistic studies on how lipids, particularly bile acids, contribute to the aetiology of SLE development, although lipid profile changes have been observed in those patients. Increasing evidence of the immune regulatory effect of lipid metabolites from other disease studies or *in vitro* studies provides us with a plausible direct elucidation of the molecular mechanisms by which the gut microbiome participates in SLE development by affecting lipid metabolism.

### Short-Chain Fatty Acids (SCFAs)

A mounting array of evidence indicates that SCFAs produced by microbes are crucial executors of the influence of intestinal bacteria on host health and disease (91, 92). SCFAs, most commonly acetate, propionate and butyrate, are the end products of dietary fibres produced by intestinal microbiota fermentation in the colon. Apart from the energy provider for colonocytes (93) and the property of promoting epithelial barrier function (94), the prominent role of SCFAs in modulating inflammation has been comprehensively reviewed elsewhere (95–97). In brief, SCFAs may serve as ligands for G-protein coupled receptors (GPCRs), including GPR41, GPR43 and GPR109A, and exert anti-inflammatory and immune regulatory effects to maintain host physiological status (98–101). The possible mechanisms by which SCFAs modulate immune homeostasis include 1) maintaining intestinal epithelial integrity and protecting against pathogen infection (102, 103); 2) improving the inflammatory environment in the host, such as reducing proinflammatory cytokine production, including IL-6, IL-12, IL-17, IFN-γ, and tumour necrosis factor-α (TNF-α), and increasing anti-inflammatory cytokine production, including TGF-β and IL-10 (96, 104–107); and 3) inducing tolerogenic and anti-inflammatory phenotypes in various immune cells, including Foxp3^+^ Treg cells, B cells and macrophages, in a GPR43-dependent manner or by histone HDAC6 and HDAC9 inhibition (108). In SLE, researchers found that the metabolic alterations associated with active disease include higher levels of 2-hydroxyisobutyrate and glutamate and lower levels of citrate, glycerol, linoleic acid, and propylparaben in serum (109). Of note, serum 2-hydroxyisobutyrate increased significantly in active patients compared with healthy individuals and inactive patients (109). Moreover, 2-hydroxyisobutyrate upregulation was also identified in the urine of SLE patients in another study published by the same group (110). As a cellular short-chain fatty acid, 2-hydroxyisobutyrate is produced mainly from microbial degradation of dietary proteins, and its presence in urine has been linked with *Faecalibacterium prausnitzii* (111), a human *Clostridia* strain well known for enhancing regulatory cell functions (112). The increased blood level of 2-hydroxyisobutyrate likely reflects altered gut microbial metabolism or is accompanied by increased gut permeability. Another recent study reported that supplementation with a high fibre-rich diet improved lupus-related disease manifestations in a Toll-like receptor 7 (TLR7)-dependent lupus-like mouse model. There were enriched gut commensal *Lactobacillus reuteri (L. reuteri)* in these lupus mice, which exacerbated lupus disease and worsened host immune mechanisms, including increasing pDCs and type I IFN gene expression. Dietary intervention with resistant starch (RS), which promotes the intestinal microbiota to ferment fibre into SCFAs, suppresses the abundance of *L. reuteri*, ameliorates lupus-like disease, downregulates type I IFN pathways and reduces lupus-related mortality (32). Fibre-derived SCFAs can also affect B cell differentiation processes that critically underpin effective T-dependent and T-independent antibody responses, leading to a reduction in autoantibodies and preventing disease manifestations in lupus-prone MRL/Faslpr/lpr and NZB/W F1 mice (113). Together, the above information indicates SCFA alterations and their beneficial immune regulatory roles and therapeutic potential in SLE.

### Tryptophan Metabolism

Tryptophan, one of the nine essential amino acids, cannot be naturally synthesized by humans, and as a result, it indispensably relies on dietary supplementation and degradation by intestinal microbiota. The metabolism of tryptophan and its metabolites is dependent on its absorption into the gut by intestinal microbiota and is closely linked to autoimmunity. Previous studies identified lower concentrations of tryptophan but higher kynurenine levels in the sera of lupus patients (114). Such findings indicate that tryptophan and the microbiota species that metabolize this amino acid may be implicated in the immune responses of SLE.

The TC mouse model expresses three lupus susceptibility loci from the lupus-prone NZM2410 mouse on the C57BL/6 background (68). The TC mouse model exhibits altered microbial communities. Faecal microbiota transplantation from aged TC mice to healthy GF B6 mice resulted in higher serum anti-dsDNA antibodies and antinuclear autoantibodies as well as the expansion of germinal centre B cells and Tfh cells. Moreover, metabolomic screening identified changes in the amount of tryptophan and its metabolites present in the subjects, including higher kynurenine levels and lower 5-HT expression in the sera and faeces of TC mice compared to B6 mice. In addition, antibiotic treatment could restore the faecal tryptophan of TC mice to normal, and dietary tryptophan restriction exerted a protective effect on lupus progression (63). A high-tryptophan diet was also found to be associated with a more severe disease phenotype and the expansion of *Prevotella, Paraprevotella*, and/or *Lactobacillus* species in TC mouse faeces and induced autoimmunity disorder after faecal transfer in GF B6 mice (**Table 2**) (63).

Multiple mechanisms have been proposed to account for the involvement of tryptophan in lupus progression. Tryptophan degradation is linked to T cell dysfunction (114) through the kynurenine pathway, which has been shown to induce indole derivatives in patients with SLE (115). Additionally, some species of *Prevotella, Paraprevotella*, or *Lactobacillus* found to be expanded in the gut of TC mice are associated with tryptophan catabolism (116–118). Many tryptophan-derived metabolites, including indole-3-aldehyde, indole-3-acetic acid, 3-methylindole, and tryptamine, are aryl hydrocarbon receptor (AhR) ligands. AhR activation could upregulate genes encoding cytokines such as IL-10 that regulate immune tolerance in lupus (119). These findings highlight the notion that modifying gut microbiota could initiate autoimmunity by manipulating tryptophan metabolism.

## Gut Microbiota, a Potential Therapeutic Target for SLE?

Due to the heterogeneity and relapsing-remitting course, SLE patients with lupus need life-long treatment to remain in remission and achieve low disease activity. Therapeutic strategies to manage lupus are highly individualized according to the clinical and/or laboratory presentation of each patient. Currently, the most widely used approaches to manage lupus include administration of glucocorticoids, hydroxychloroquine, immunosuppressive (IS) drugs, and biological agents to improve long-term patient outcomes (120). Notably, there are substantial side effects associated with available treatments and other negative outcomes, such as inducing an infectious rate. These drawbacks make it essential to identify new interventions that are effective, such as targeted drugs, or ways to reduce adverse reactions.

### Treatment Strategy Based on Diet-Induced Microbiota Adjustment

Dietary interventions have long been considered feasible and economical auxiliary options to improve symptoms in autoimmune diseases such as RA. There are some encouraging results obtained from clinical and laboratory studies, for example, using α-glucosidase inhibitors or high fibre-rich diets for RA therapy (121, 122). All of the mechanistic investigations point to the involvement of gut microbiota in the above treatments. These findings suggest the possibility that dietary-mediated manipulation of gut microbiota would be a plausible preventive strategy for individuals susceptible to SLE. In fact, acidic water changes the composition of the gut microbiota by increasing *Firmicutes* and reducing *Bacteroidetes in* spp. in favour of renal protection, as reported in an experimental study (59). Another study suggests beneficial effects of vitamin A on autoimmune responses, as vitamin An improves lupus symptoms by promoting *lactobacilli* expansion (60). Recently, Zegarra-Ruiz *et al.* reported that resistant starch diet-derived short-chain fatty acids could suppress *L. reuteri*, a newly identified pathogen in the pathogenesis of SLE. A starch diet, therefore, is capable of ameliorating lupus-related mortality and manifestations by decreasing pDCs and suppressing the type I IFN pathway (32).

### Treatment by Probiotics/Prebiotics

The use of probiotics or prebiotics is another way to improve microbial-associated disorders. Long-term use of probiotics can regulate inflammatory status and reduce autoantibody production, thereby lessening lupus severity (123). López *et al.* revealed that microbiota isolated from SLE patient stool samples promoted lymphocyte activation and naïve CD4^+^ lymphocyte differentiation towards the Th17 subset compared with that isolated from healthy controls. Interestingly, when 5, 10 or 30% of SLE gut microbiota were replaced with the same proportions of *Bifidobacterium bifidum* LMG13195 (Bb) or a mixture of two *Clostridia* strains (CI: *Ruminococcus obeum* DSM25238 and *Blautia coccoides* DSM935), which are known for Treg-inducing effects, Bb and CI significantly reduced CD4^+^ lymphocyte over-activation and Th17/Th1 balance, respectively (124). Mike *et al.* showed that ingestion of a diet containing *Lactobacillus casei* (LC) from the weaning period could prolong the lifespan of MRL/lpr mice and prevent the expansion of B220+ T cells in the spleen and mesenteric lymph nodes. Furthermore, intraperitoneal injection of LC into MRL/lpr mice also significantly impaired the proliferative capacities of splenocytes and the accumulation of B220^+^CD4^-^CD8^-^ T cells with increasing I-A^–^ macrophages and macrophage colony-forming cells (M-CFCs) (125).

Overall, although research on SLE therapy based on gut microbiota regulation is limited, the existing evidence points to a plausible strategy for SLE treatment. Of course, the underlying mechanisms implicated in the specific diet or prebiotics/probiotics’ improving effects on SLE remain to be elucidated, and further systematic research is warranted.

## Conclusion

The involvement of mucosal microbiomes in the development of SLE has gained increasing attention from rheumatologists and immunologists with the development of new techniques and new advances. Although some recent studies have identified several pathobionts and pathways that are related to local or systemic inflammation processes and immune system dysregulation in animal lupus models (**Table 2**), it remains to be determined how consistent they are among different patient populations and which microbial antigens drive immune intolerance to produce several lupus-related autoantibodies. Additionally, sex-specific differences in intestinal bacteria are observed in rodent lupus models (**Table 2**) (64), but the reason behind these differences is not fully understood. The results of association studies should be interpreted with caution, and a larger array of techniques, including a wider use of longitudinal studies and multi-omics data, will be required to solidify the above questions. Over the last decade, it has become increasingly apparent that the gut microbiota interacts with the host across great distances within the body by producing metabolites. However, there are many unknowns about the clear-cut mechanisms by which microbiota-derived metabolites or co-metabolites (such as AhR ligands) affect the immune cell phenotype and function in the process of SLE development. Filling this gap will definitely advance our understanding of the way in which intestinal microbiota participate in the aetiology of SLE. Recent studies highlight innovative microbiota-based therapeutic strategies, including dietary intervention, probiotic/prebiotic supplementation and FMT, which show therapeutic benefits in IBD, obesity, metabolic syndrome and depressive disorder in the clinic (126–130). Thus far, the results from studies of microbiota-based therapy in lupus animal models seem to be positive and provide us with a plausible possibility to develop an innovative SLE treatment method in the future.

## Author Contributions

LZ and PQ drafted the manuscript. HY and YW collected related literature. YBL and YL supervised the work and revised the manuscript. All authors contributed to the article and approved the submitted version.

## Funding

This research was funded by the National Natural Science Foundation of China (No. 81770101, 81403041), Outstanding interdisciplinary project of West China Hospital, Sichuan University (No. ZYJC18024), 1.3.5 project for disciplines of excellence, West China Hospital, Sichuan University (Project no. ZYGD18015). Scientific application and foundation project of Science and Technology Department of Sichuan Province (No. 2020YJ0021), Scientific research project of Health Commission of Sichuan province (No. 20PJ050).

## Conflict of Interest

The authors declare that the research was conducted in the absence of any commercial or financial relationships that could be construed as a potential conflict of interest.
